# Data management plan for a community-level study of the hidden burden of cutaneous leishmaniasis in Colombia

**DOI:** 10.1186/s13104-021-05618-4

**Published:** 2021-05-31

**Authors:** Oscar Javier Oviedo Sarmiento, María del Mar Castro, Yenifer Orobio Lerma, Leonardo Vargas Bernal, Andrés Navarro, Neal D. E. Alexander

**Affiliations:** 1 CIDEIM, Calle 18 #122-135 Campus de la Universidad Icesi (Edificio O-CIDEIM), Cali, 760031 Colombia; 2 Universidad Icesi Grupo de investigación en informática y telecomunicaciones i2t, Calle 18 #122-135, Cali, 760031 Colombia

**Keywords:** MHealth, Data management plan, Cutaneous leishmaniasis, Under-reporting

## Abstract

**Objectives:**

Cutaneous leishmaniasis is a vector-borne parasitic disease whose lasting scars can cause stigmatization and depressive symptoms. It is endemic in remote rural areas and its incidence is under-reported, while the effectiveness, as opposed to efficacy, of its treatments is largely unknown. Here we present the data management plan (DMP) of a project which includes mHealth tools to address these knowledge gaps in Colombia. The objectives of the DMP are to specify the tools and procedures for data collection, data transfer, data entry, creation of analysis dataset, monitoring and archiving.

**Results:**

The DMP includes data from two mobile apps: one implements a clinical prediction rule, and the other is for follow-up and treatment of confirmed cases. A desktop interface integrates these data and facilitates their linkage with other sources which include routine surveillance as well as paper and electronic case report forms. Multiple user and programming interfaces are used, as well as multiple relational and non-relational database engines. This DMP describes the successful integration of heterogeneous data sources and technologies. However the complexity of the project meant that the DMP took longer to develop than expected. We describe lessons learned which could be useful for future mHealth projects.

**Supplementary Information:**

The online version contains supplementary material available at 10.1186/s13104-021-05618-4.

## Introduction

Cutaneous leishmaniasis (CL) is a parasitic disease transmitted by sandfly vectors [1, 2]. Lesions typically occur in areas which are exposed to sandfly bites, and the resulting scars can cause stigmatization, anxiety, and depressive symptoms [3]. In Colombia, most cases of CL occur in rural areas, with the most common parasite species being *Leishmania* (*Viannia*) *panamensis* and *L.* (*V.*) *braziliensis* [4], and the most important vectors including *Psychodopygus panamensis*, *P. amazonensis* and *Pintomyia* (*Pifanomyia*) *longiflocosa* [5].

Control of CL in Colombia, and in the Americas more generally, largely relies on passive case detection and ambulatory treatment without comprehensive follow-up, hence there is little information on clinical response and adverse drug reactions [6, 7], with under-reporting being particularly high in rural areas. In Colombia, the SIVIGILA surveillance system records CL at the level of municipality [8], although the incidence has been estimated to be under-reported by a factor of 2.8–4.6 [2].

In order to address these knowledge gaps, in Colombia we are carrying out a project titled “Estimating the hidden burden of cutaneous leishmaniasis by predictive risk mapping and quantifying under-reporting and effectiveness of standard-of-care treatment”. In order to use community-level ascertainment of CL cases, this project utilizes mHealth tools, specifically mobile apps. Moreover, as well as custom databases designed for the study, we use data with heterogeneous formats from the public health surveillance system and individual health facilities. Our responses to these challenges are described in the current data management plan (DMP).

## Main text

This DMP follows the structure of CIDEIM’s standard DMP format which, in turn, used the concepts of Prokscha [9]. Since the first version of the format in 2013, the evolution of information systems and communication networks has required data models to accommodate decentralized multiplatform schemes, which present new challenges to data security and audit. Hence the DMP format has evolved to continue providing traceability based on the minimum audit elements required to guarantee validity of clinical study data.

### Objectives and epidemiological design of the CL study

The overall aim of the project is to address knowledge gaps in the targeting and evaluation of interventions for the control of cutaneous leishmaniasis. Additional file 1: Figure S1 is an overview of the activities and objectives of the project.

Objective 1: estimate under-reporting and under-ascertainment of CL using mHealth assisted active case detection, chain-referral sampling, and community-based surveillance. Epidemiological under-estimation has two components: under-reporting and under-ascertainment [10]: the former occurs when patients affected by notifiable diseases visit health facilities, are diagnosed, but are not reported to the surveillance system; the latter refers to the number of infected individuals who are affected but not even diagnosed. This project will estimate under-reporting via local registers of health system attendance and health facility laboratory reports. We will estimate under-ascertainment by a combination of complementary methods: chain-referral sampling, community-based surveillance, and active surveillance by public health teams and community leaders with the aid of mHealth apps.

Objective 2: develop a model, based on remote sensing and vector niches, for predicting risk of cutaneous leishmaniasis, and estimate its ability to identify high-risk sites We will combine spatial statistical [11] and species niche modelling [12] approaches to identify, at a small scale, areas with elevated risk of CL. Output from the niche model will be included in the spatial statistical model and its predictions compared with the under-ascertainment data from Objective 1.

Objective 3: estimate the effectiveness of the standard treatment for CL in three municipalities, supported by the community-based use of mHealth tools. We will enhance an existing Android application to facilitate measurement of compliance, adverse drug reactions and final therapeutic response (cure or failure), the last of these via lesion photographs to be assessed remotely.

### Data collection tools and procedures

We previously developed two mobile applications (“apps”). The first is called Guaral RPC, and implements a clinical prediction rule (*regla de predicción clínica* in Spanish) for screening possible cases of CL [13, 14]. The second is called Guaral +ST, and is for the follow-up and treatment (*seguimiento y tratamiento*) of confirmed cases. Data from the latter include images. We will describe the tools and procedures by objective: first those primarily concerned with human cases (Objectives 1 and 3), and then that which is more concerned with the insect vectors (Objective 2).

### Tools and procedures for Objectives 1 and 3

**Fig. 1:**
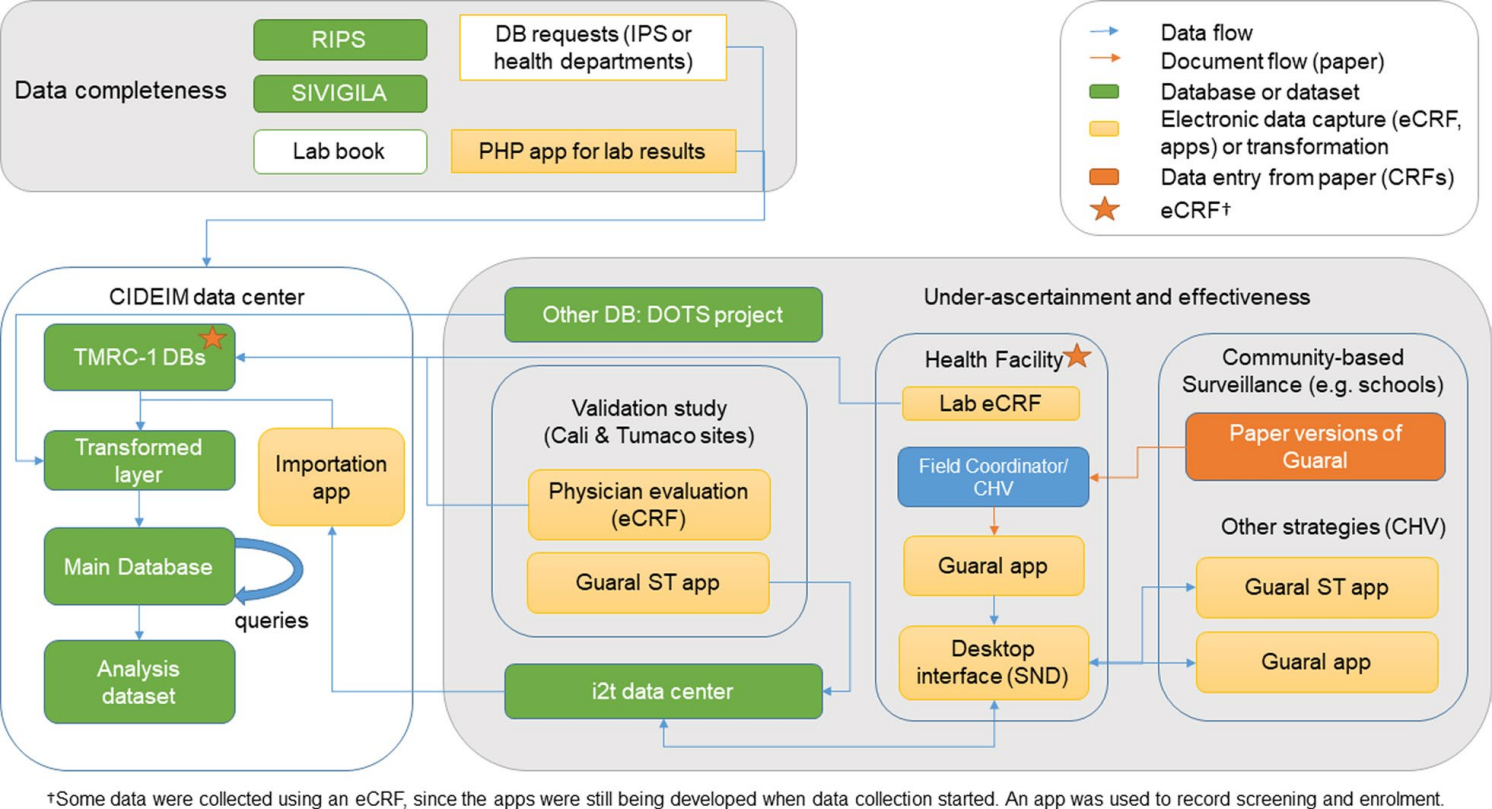
Data flow for Objectives 1 and 3. The mHealth apps are shown in the box for under-ascertainment and effectiveness (bottom right). The various data sources are brought together in the CIDEIM data center (bottom left). Although all the processes shown are part of the TMRC project, some collection methods, such as the mHealth apps, pre-date this. The “TMRC1 DBs” in the figure are databases which were created specifically for the project. Enrollment is a more general process which is necessary for all CIDEIM projects and is not included here. See also the List of Abbreviations

For under-reporting (data completeness), we will compare multiple data sources including: Registros Individuales de Prestación de Servicios de Salud (RIPS), SIVIGILA surveillance records, and clinical laboratory results from residents of the selected townships from health facilities (Instituciones Prestadoras de Salud, IPS). The RIPS and SIVIGILA databases (in ASCII flat files) will be requested from the Departmental or Municipal health departments (in Colombia the first-level administrative division is the Department, and the second-level is the Municipality). The data flow is shown in Fig. 1. A RIPS database comprises ten ASCII files [15], each with one comma-delimited record per line (Table 1). From SIVIGILA we will request notifications of the “basic data” sheet and event codes 420 (cutaneous leishmaniasis), 430 (mucosal leishmaniasis) and 440 (visceral leishmaniasis).

**Table 1:** Component files of a RIPS (Individual Health Service Records) dataset

CT	Control (master file)*
AF	Transactions
US	Health service users*
AC	Consultations and diagnoses (ICD-10 code)*
AP	Procedures (CUPS code)*
AU	Emergency admissions under observation
AH	Hospitalizations
AN	Newborns
AM	Medication
AT	Other services

Files which are most relevant to the current project are indicated by *

Laboratory data from health facilities (IPS) may be entered in CIDEIM based on photographs of the corresponding laboratory books. This process uses PHPMaker [16], a tool for automating applications, which scans the table structure (schema) and generates user interfaces to the basic CRUD operations (Create, Read, Update y Delete) [17], guaranteeing traceability (audit trail). It also allows access control in the form of user names and encrypted passwords. This tool produces an application in PHP and HTML version 5, with the model-view-controller (MVC) architecture [18].

Estimation of under-ascertainment and effectiveness will use the mobile apps Guaral RPC and Guaral +ST (Fig. 1). Guaral RPC will be mainly used for community-based surveillance, since it handles identifying information of the participants, and variables for presumptive CL diagnosis.

Given the limited internet access in these areas, the data are stored in a local SQLite database in the app. When the app detects connectivity, it tries to synchronize, sending the locally stored data, with encryption, to the i2t data center at Icesi University, and bringing data of any new patients which have been assigned to community health volunteers (CHV) in the SND system (see “Data transfer” below). Photographs taken by the CHV are kept in the phone, subject to storage capacity. A substudy, known as the validation study, assesses the accuracy of Guaral +ST in terms of remotely determining the therapeutic response to CL treatments. Data on some patients from a previous leishmaniasis project (known as DOTS, or Directly Observed Treatment, Short Course) will be included as historical controls.

### Tools and procedures for Objective 2

The data management procedures for this objective are more straightforward than for those of the other objectives and independent of the latter (Additional file 1: Figure S1). It uses remote sensing data, such as satellite images from the MODIS repository (https://earthexplorer.usgs.gov/) and WorldClim (http://worldclim.org/version2), as well as published and unpublished data on field catches of the vector species. These data were imported into ArcGIS for analysis. The project also carried out its own field catches to validate the predictions, and these data were recorded on paper forms and then entered into Microsoft Excel.

Table 2 lists the source documents for each CRF and other data collection tool. In some cases the collection tool will be its own source document.

**Table 2:** Source documents

CRF or other data collection tool	Where collected	Source document
Guaral RPC: presumptive diagnosis, and initialization of the patient in the system.	Field or health facility	Guaral RPC, or possibly a paper version for community-based surveillance
Guaral +ST: adverse events and treatment follow-up	Field	Guaral +ST
Guaral +ST: photographs of treatment follow-up	Field or health facility	Guaral +ST
Treatment follow-up card	Field or health facility	Treatment follow-up card (used only if the Guaral +ST app is not available)
SND desktop interface: record of the prescribed treatment	Health facility	Medical record
PHP application for laboratory results	Health facility	Laboratory book
SND desktop interface: allocation of CHV to follow-up	Health facility	Desktop SND interface
Laboratory results eCRF	CIDEIM (Cali)	Medical record (Histoweb)
Validation study CRF	CIDEIM (Cali)	Validation study CRF
Vector (entomology) CRF	Field	Vector CRF
Screening and enrolment desktop app	Health facility	Screening and enrolment desktop app
DOTS project database	Existing data from DOTS project	DOTS CRF

### Data transfer

The data flow is shown in Fig. 1. Paper CRFs will be temporarily stored at the field sites, with access limited to study staff. For data entry and archiving, the CRFs will be sent by certified mail to CIDEIM, where receipt of each batch will be documented.

The SND desktop interface to the mHealth apps is a web application created in ASP.NET Core. It has five components, deployed independently in Docker containers: the Guaral RPC API, the Guaral+ST API, the Web UI, the non-relational database and the relational database. It allows field coordinators to register projects and CHV, specify treatments (assignment), inspect patients’ information and make assessments. Data from the CHV can be accessed by researchers via this interface, which uses web services to connect to the i2t data center’s server. A process called assignment, performed in the SND web interface, allows the field coordinator to select a CHV to follow up a given CL cases, based on logistic considerations. This assignment also moves the patient’s information from one container (Guaral RPC) to another (Guaral +ST).

The synchronized information is stored in a group of relational (PostgreSQL) and non-relational (MongoDB) databases, and the photos are sent to an instance of MinIO, which is an open source, high performance object store. The SND interface also allows data to be exported, in accordance with a pre-defined data dictionary.

### Data entry

The data from paper CRFs will be double-entered. Discrepancy (query) reports will be sent to the investigator for resolution, using a standard form. Single data entry is used for the eCRFs and mobile apps. To guarantee the quality of the data, multiple strategies were employed prior to the design of the data collection instruments and after their use. eCRFs were pilot-tested and modified accordingly. The study team will be trained in the handling of these instruments and, finally, clinical monitoring will be carried out (see below) to guarantee the quality of the data.

### Creation of analysis dataset

An application has been developed to import the mHealth data by decompressing an export from the SND platform, carrying out ETL (Extract, Transform and Load) operations. This application uses SQL Server Integration Services (SSIS) which is part of the Microsoft SQL Server 2008 database engine. This process allows patients to be selected globally or individually from the SND platform. These data will be part of the main database comprising the sources shown in Fig. 1 and Table 2.

### Clinical monitoring

The clinical monitor will carry out quality control of the paper and electronic records according to the study’s monitoring plan. The findings are recorded on a standard report form and passed to the study’s project manager for review and, if necessary, resolved in the project database by liaising with the data manager.

### Audit trail

Audit trail is maintained throughout the data flow by capturing information on the date, time and user who makes the modification. Procedures were designed to also capture the origin of the data by identifying the MAC addresses of the devices used, and their location by means of IP addresses.

### Archiving of CRFs and electronic data

Paper CRFs will be stored in the central archive of CIDEIM in Cali, except during data entry. eCRF data will be stored by the Unidad de Investigación en Epidemiología y Bioestadística (UIEB) of CIDEIM, and backed up according to an existing SOP. The information will be kept in an active file for three years after the study closes, and then in the institutional inactive file for a maximum of 20 years.

### Discussion

To our knowledge, this is the first peer-reviewed publication of a data management plan for an mHealth project. The integration of multiple data sources, including mHealth apps and paper and electronic CRFs, and the collaboration between multiple academic and healthcare entities, complicated data management and meant that the development of the DMP took longer than expected. This experience overlaps with the implementation research perspective of Meyer et al. [19], who found that “complex data structures impeded the development and execution of a data management plan that would allow for articulation of goals and provide timely feedback to study staff, CHWs, and participants”. Interoperability was also one of the principal challenges in implementing mHealth identified by Gurupur and Wan [20].

Another challenge is maintaining the integrity of the data as they flow through the various processes. Data are sourced from different parts of the country, and are subject to different network conditions and device connectivity. This led to the improvement of the data validation and auditing processes to ensure the integrity and secure handling of participant data.

Exacting technical requirements, such as those noted above, have previously been identified as an important barrier to the use of mHealth [21]. This characteristic of mHealth is in tension with its philosophy of making diagnosis and treatment more readily available in resource-limited settings [19]. In the current project, the apps are not directly integrated with existing health service systems and, although this would have further complicated the data management, such integration does favour the lasting impact of such innovations [22].

In conclusion, we have established a working data management plan for this complex project, and one of the main lessons learned is the need to allow sufficient time to integrate the components. Future projects would also benefited from greater integration with health systems, a process which could benefit from techniques of implementation research.

### Limitations

- The complexity of the DMP meant it took longer than planed to develop.
- The mobile apps are not integrated with existing public health tools.

## Supplementary Information

**Additional file 1: Fig. S1.** Schematic diagram of the project’s activities and objectives. Objectives 1 and 3 include the mHealth apps and are the most challenging in terms of data management.

## Data Availability

The current report contains no human data. We will follow data sharing policies of the funder (NIH) and scientific journals, subject to authorization by the CIDEIM ethics committee. In addition, we will consider written requests for data access from any practising scientist affiliated to a research institution. Subject to agreement by the IRBs of the participating institutions, we will send the data to the applicant by email. We will not seek to impose restrictions on the purposes for which the data may be used. However, we will impose the following conditions. (1) the applicant(s) may not pass the data on to any person other than any co-applicants named in the original request, and must implement security measures to prevent such access. (2) the applicant(s) may not seek to identify individuals by data-mining techniques or otherwise. Data from the project will be stored in an active archive for three years following closure of the study, after which files years. Universidad Icesi owns copyright in the mobile apps (Guaral RPC and Guaral +ST) and in the SND system.

## Abbreviations

API: Application programming interface
ASCII: American Standard Code for Information Interchange
CIDEIM: Centro Internacional de Entrenamiento e Investigaciones Medicas (International Center for Medical Research and Training)
CL: Cutaneous leishmaniasis
CHV: Community health volunteer
CRF: Case report form (paper)
CRUD: Create, read, update and delete
CUPS: *codificación única de procedimientos* (unique procedure coding)
DB: Database
DMP: Data management plan
DOTS project: Previous CIDEIM project on access to leishmaniasis diagnosis and treatment. DOTS stands for Directly Observed Treatment, Short Course.
EAPB: Entidades Administradoras de Planes de Beneficios (Entities Administrating Benefit Plans)
eCRF: Electronic case report form
ETL: Extract, transform and load
FVL: Fundación Valle del Lili, Cali
Guaral RPC: Mobile app which implements a clinical prediction rule (*regla de predicción clínica*, RPC)
Guaral +ST: Mobile app for follow-up and treatment (*seguimiento y tratamiento*, ST) of cutaneous leishmaniasis patients
Histoweb: CIDEIM in-house system for electronic medical records
HTML: Hypertext Markup Language
ICD-10: International Classification of Diseases, Tenth Revision
IP address: Internet Protocol address
IPS: Institución Prestadora de Salud (Institutional Health Service Provider)
MAC address: Media access control address
mHealth: Mobile health
MODIS: Moderate Resolution Imaging Spectroradiometer
MVC: Model-view-controller
NIH: National Institutes of Health, United States of America
PHP: A scripting language. PHP originally stood for Personal Home Page, but now stands for PHP: Hypertext Preprocessor.
RIPS: Registros Individuales de Prestación de Servicios (Individual Health Service Records)
SIVIGILA: Sistema Nacional de Vigilancia en Salud Pública (National Public Health Surveillance System)
SND: System for Neurological Diseases (this has now been expanded to other conditions)
SOP: Standard operating procedure
SQL: Structured Query Language
SSIC: SQL Server Integration Services
TMRC: Tropical Medicine Research Center: an NIH funding scheme and a name used for the current project
UI: User interface
UIEB: Unidad de Investigación en Epidemiología y Bioestadística (Epidemiology and Biostatistics Research Unit of CIDEIM)

## References

[1] Karimkhani C, Wanga V, Coffeng LE, Naghavi P, Dellavalle RP, Naghavi M (2016). Global burden of cutaneous leishmaniasis: a cross-sectional analysis from the global burden of disease study 2013. Lancet Infect Dis.

[2] Alvar J, Velez ID, Bern C, Herrero M, Desjeux P, Cano J, Jannin J, den Boer M (2012). WHO Leishmaniasis Control Team: Leishmaniasis worldwide and global estimates of its incidence. PLoS ONE.

[3] Yanik M, Gurel MS, Simsek Z, Kati M (2004). The psychological impact of cutaneous leishmaniasis. Clin Exp Dermatol.

[4] Rodríguez-Barraquer I, Góngora R, Prager M, Pacheco R, Montero LM, Navas A, Ferro C, Miranda MC, Saravia NG (2008). Etiologic agent of an epidemic of cutaneous leishmaniasis in Tolima, Colombia. Am J Trop Med Hyg.

[5] Ferro C, Lopez M, Fuya P, Lugo L, Cordovez JM, Gonzalez C (2015). Spatial distribution of sand fly vectors and eco-epidemiology of cutaneous leishmaniasis transmission in Colombia. PLoS ONE.

[6] Pan American Health Organization. Leishmaniases: epidemiological report of the Americas. Washington, D.C.: Report; 2014.

[7] Pan American Health Organization. Leishmaniases: epidemiological Report of the Americas. Washington, D.C.: Report; 2015.

[8] Agudelo Chivatá, N.J.: Informe de Evento Leishmaniasis Cutánea, Periodo Epidemiológico XIII. Colombia, 2019. Report; 2019.

[9] Prokscha S (1999). Practical guide to clinical data management.

[10] European Centre for Disease Prevention and Control. Data quality monitoring and surveillance system evaluation—a handbook of methods and applications. Stockholm: ECDC; 2014.

[11] Lawson A (2013). Bayesian disease mapping: hierarchical modeling in spatial epidemiology. Interdisciplinary statistics.

[12] Phillips SJ, Anderson RP, Schapire RE (2006). Maximum entropy modeling of species geographic distributions. Ecol Model.

[13] Weigle KA, Escobar M, Arias AL, Martinez F, Rojas C (1993). A clinical prediction rule for American cutaneous leishmaniasis in Colombia. Int J Epidemiol.

[14] Rubiano L, Alexander NDE, Castillo RM, Martínez AJ, García Luna JA, Arango JD, Vargas L, Madriñán P, Hurtado L-R, Orobio Y, Rojas CA, del Corral H, Navarro A, Saravia NG, Aronoff-Spencer E (2021). Adaptation and performance of a mobile application for early detection of cutaneous leishmaniasis. PLoS Negl Trop Dis.

[15] Oficina de Tecnología de la Información y la Comunicación: Lineamiento Técnico para el Registro y Envío de los Datos del Registro Individual de Prestaciones de Salud – RIPS, desde las Instituciones Prestadoras de Servicios de Salud a las EAPB. Report, Ministerio de Salud; 2017.

[16] e.World Technology Limited: PHPMaker; 2020. https://phpmaker.dev/. Accessed 10 Nov 2020.

[17] Gorman BL. Data access (create, read, update, delete). In: Practical entity framework; 2020, pp. 245–69. Apress, Berkeley, CA.

[18] Lucassen JM, Maes SH. MVC (model-view-controller) based multi-modal authoring tool and development environment. Patent Number US 6996800 B2; 2006.

[19] Meyer AJ, Armstrong-Hough M, Babirye D, Mark D, Turimumahoro P, Ayakaka I, Haberer JE, Katamba A, Davis JL (2020). Implementing mHealth interventions in a resource-constrained setting: case study from Uganda. JMIR Mhealth Uhealth.

[20] Gurupur VP, Wan TTH (2017). Challenges in implementing mHealth interventions: a technical perspective. Mhealth.

[21] Kruse C, Betancourt J, Ortiz S, Valdes Luna SM, Bamrah IK, Segovia N (2019). Barriers to the use of mobile health in improving health outcomes in developing countries: systematic review. J Med Internet Res.

[22] Dharmayat KI, Tran T, Hardy V, Chirambo BG, Thompson MJ, Ide N, Carlsson S, Andersson B, O’Donoghue JM, Mastellos N (2019). Sustainability of "mHealth" interventions in sub-Saharan Africa: a stakeholder analysis of an electronic community case management project in Malawi. Malawi Med J.

