# Supplementary figures and images for: Data management plan for a community-level study of the hidden burden of cutaneous leishmaniasis in Colombia
Source: BMC Res Notes. 2021 May 31;14:213. doi: 10.1186/s13104-021-05618-4 (PMC8165780; doi:10.1186/s13104-021-05618-4)

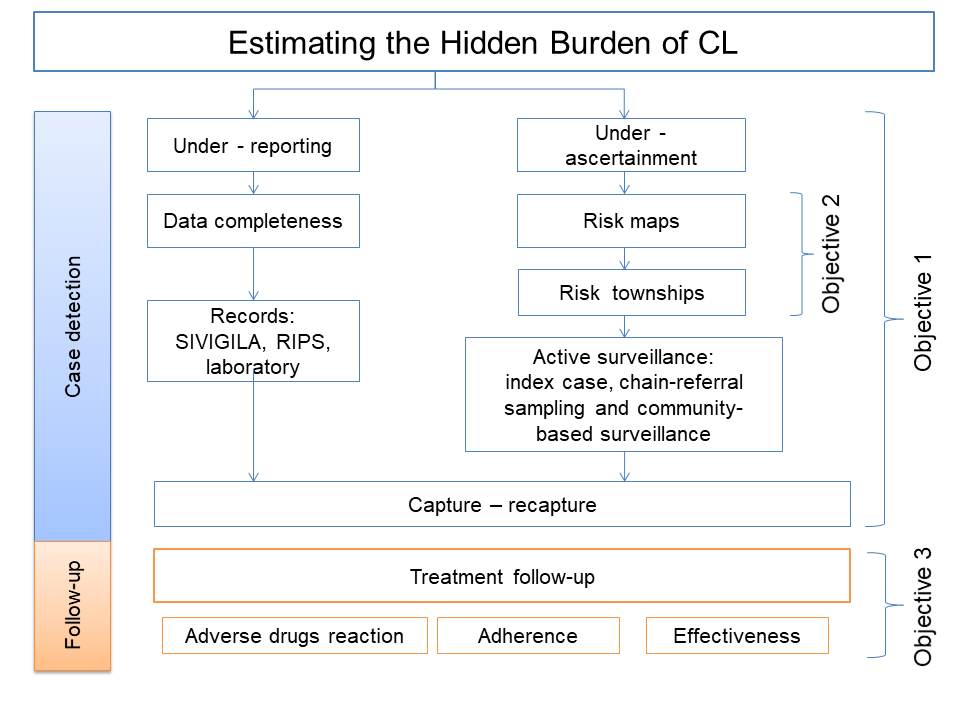

Supplement: Supplementary file 1 — Additional file 1: Fig. S1. Schematic diagram of the project’s activities and objectives. Objectives 1 and 3 include the mHealth apps and are the most challenging in terms of data management. [file 13104_2021_5618_MOESM1_ESM.png]
